# Supplementary material for: One health genomic surveillance and response to a university-based outbreak of the SARS-CoV-2 Delta AY.25 lineage, Arizona, 2021
Source: PLoS One. 2022 Oct 31;17(10):e0272830. doi: 10.1371/journal.pone.0272830 (PMC9621446; doi:10.1371/journal.pone.0272830)
Supplement: S1 Table — (PDF) [file pone.0272830.s001.pdf]

GISAID Accession ID	Date of Collection	Affiliation
EPI_ISL_4052586	8/30/2021	University
EPI_ISL_4052499	8/31/2021	University
EPI_ISL_4090897	9/2/2021	University
EPI_ISL_4090972	9/4/2021	Community
EPI_ISL_4410585	9/8/2021	Community
EPI_ISL_4410573	9/8/2021	University
EPI_ISL_4410637	9/9/2021	University
EPI_ISL_4410586	9/9/2021	University
EPI_ISL_4469488	9/10/2021	University
EPI_ISL_4469501	9/10/2021	University
EPI_ISL_4410427	9/11/2021	University
EPI_ISL_4469400	9/13/2021	University
EPI_ISL_4469357	9/13/2021	University
EPI_ISL_4469509	9/13/2021	University
EPI_ISL_4469428	9/13/2021	Community
EPI_ISL_5079080	9/13/2021	Community
EPI_ISL_4469301	9/13/2021	University
EPI_ISL_4469334	9/13/2021	University
EPI_ISL_4469441	9/13/2021	University
EPI_ISL_4469498	9/14/2021	University
EPI_ISL_4469280	9/14/2021	University
EPI_ISL_4469526	9/14/2021	Community
EPI_ISL_4469332	9/14/2021	Community
EPI_ISL_4469518	9/14/2021	University
EPI_ISL_4469500	9/14/2021	University
EPI_ISL_4469289	9/14/2021	University
EPI_ISL_4469286	9/14/2021	University
EPI_ISL_4469304	9/14/2021	University
EPI_ISL_4469281	9/14/2021	University
EPI_ISL_4469453	9/14/2021	University
EPI_ISL_4513287	9/15/2021	University
EPI_ISL_4513149	9/15/2021	University
EPI_ISL_4513130	9/15/2021	Community
EPI_ISL_4513025	9/15/2021	University
EPI_ISL_4513046	9/15/2021	University
EPI_ISL_4513029	9/15/2021	University
EPI_ISL_4513160	9/16/2021	Community
EPI_ISL_4512987	9/16/2021	University
EPI_ISL_4513145	9/16/2021	University
EPI_ISL_4513048	9/16/2021	University
EPI_ISL_4513178	9/16/2021	University
EPI_ISL_4513180	9/16/2021	University

EPI_ISL_4513100	9/16/2021	University
EPI_ISL_4513063	9/16/2021	University
EPI_ISL_4513106	9/17/2021	Community
EPI_ISL_4513193	9/17/2021	Community
EPI_ISL_4513127	9/17/2021	University
EPI_ISL_4513310	9/17/2021	University
EPI_ISL_4513077	9/17/2021	University
EPI_ISL_4513173	9/17/2021	University
EPI_ISL_4513097	9/17/2021	University
EPI_ISL_4513224	9/17/2021	Community
EPI_ISL_4513062	9/18/2021	University
EPI_ISL_6254573	9/18/2021	Unknown
EPI_ISL_4557360	9/20/2021	University
EPI_ISL_4557327	9/20/2021	University
EPI_ISL_5165658	9/20/2021	Community
EPI_ISL_4557445	9/20/2021	University
EPI_ISL_4557372	9/20/2021	University
EPI_ISL_4557490	9/20/2021	University
EPI_ISL_4557368	9/21/2021	University
EPI_ISL_4557370	9/21/2021	University
EPI_ISL_4557379	9/21/2021	Community
EPI_ISL_4557351	9/21/2021	Community
EPI_ISL_4557354	9/21/2021	University
EPI_ISL_4557371	9/21/2021	University
EPI_ISL_4557355	9/21/2021	University
EPI_ISL_4557373	9/21/2021	University
EPI_ISL_4557346	9/21/2021	University
EPI_ISL_4557358	9/21/2021	University
EPI_ISL_4744364	9/22/2021	University
EPI_ISL_4744292	9/22/2021	University
EPI_ISL_4744370	9/22/2021	University
EPI_ISL_4744352	9/22/2021	University
EPI_ISL_4744392	9/22/2021	University
EPI_ISL_4744377	9/22/2021	University
EPI_ISL_4744363	9/22/2021	University
EPI_ISL_4744353	9/22/2021	University
EPI_ISL_4744384	9/22/2021	University
EPI_ISL_4744391	9/22/2021	University
EPI_ISL_4744341	9/22/2021	University
EPI_ISL_4744367	9/22/2021	Community
EPI_ISL_4744357	9/22/2021	University
EPI_ISL_4744351	9/22/2021	University
EPI_ISL_4744210	9/22/2021	University

EPI_ISL_4744175	9/22/2021	University
EPI_ISL_4744239	9/22/2021	University
EPI_ISL_4744344	9/22/2021	University
EPI_ISL_4744337	9/22/2021	University
EPI_ISL_4744141	9/23/2021	University
EPI_ISL_4744360	9/23/2021	University
EPI_ISL_4744309	9/23/2021	University
EPI_ISL_4744390	9/23/2021	University
EPI_ISL_4744355	9/23/2021	University
EPI_ISL_4744362	9/23/2021	University
EPI_ISL_7379237	9/23/2021	Community
EPI_ISL_4744125	9/23/2021	Community
EPI_ISL_4744358	9/23/2021	Community
EPI_ISL_4744331	9/23/2021	University
EPI_ISL_4744354	9/23/2021	University
EPI_ISL_4744140	9/23/2021	University
EPI_ISL_4744396	9/23/2021	University
EPI_ISL_4744366	9/23/2021	University
EPI_ISL_4744203	9/24/2021	University
EPI_ISL_4744211	9/24/2021	University
EPI_ISL_4744192	9/24/2021	University
EPI_ISL_4744142	9/24/2021	University
EPI_ISL_4744330	9/24/2021	University
EPI_ISL_4744395	9/24/2021	University
EPI_ISL_4744348	9/24/2021	University
EPI_ISL_4744342	9/24/2021	University
EPI_ISL_4744381	9/24/2021	University
EPI_ISL_4744343	9/24/2021	University
EPI_ISL_8365525	9/24/2021	Community
EPI_ISL_4744356	9/24/2021	Community
EPI_ISL_4744394	9/24/2021	Community
EPI_ISL_4744197	9/24/2021	University
EPI_ISL_4744131	9/24/2021	University
EPI_ISL_4744365	9/24/2021	University
EPI_ISL_4968359	9/24/2021	University
EPI_ISL_4744371	9/24/2021	University
EPI_ISL_4744368	9/24/2021	University
EPI_ISL_4968136	9/25/2021	University
EPI_ISL_7379252	9/25/2021	Community
EPI_ISL_4968090	9/25/2021	University
EPI_ISL_4968335	9/25/2021	University
EPI_ISL_4968267	9/25/2021	University
EPI_ISL_5170422	9/26/2021	Community

EPI_ISL_4968114	9/27/2021	University
EPI_ISL_4968208	9/27/2021	University
EPI_ISL_4968279	9/27/2021	University
EPI_ISL_4968143	9/27/2021	University
EPI_ISL_7379249	9/27/2021	Community
EPI_ISL_7379250	9/27/2021	Community
EPI_ISL_5170630	9/27/2021	Community
EPI_ISL_4902130	9/27/2021	Community
EPI_ISL_4968285	9/27/2021	Community
EPI_ISL_4902159	9/27/2021	Community
EPI_ISL_4968225	9/27/2021	University
EPI_ISL_4968349	9/27/2021	University
EPI_ISL_4968302	9/27/2021	University
EPI_ISL_4968348	9/27/2021	University
EPI_ISL_4968230	9/27/2021	University
EPI_ISL_4968346	9/27/2021	University
EPI_ISL_4968381	9/27/2021	University
EPI_ISL_4968185	9/27/2021	University
EPI_ISL_4968198	9/27/2021	University
EPI_ISL_5059500	9/28/2021	University
EPI_ISL_4968352	9/28/2021	University
EPI_ISL_4968089	9/28/2021	University
EPI_ISL_4968398	9/28/2021	Community
EPI_ISL_4968241	9/28/2021	University
EPI_ISL_5059633	9/28/2021	University
EPI_ISL_4968170	9/28/2021	University
EPI_ISL_5059661	9/28/2021	University
EPI_ISL_5059118	9/28/2021	Community
EPI_ISL_5059634	9/28/2021	University
EPI_ISL_4968328	9/28/2021	University
EPI_ISL_5059665	9/28/2021	University
EPI_ISL_5059156	9/29/2021	University
EPI_ISL_5059694	9/29/2021	University
EPI_ISL_5082888	9/29/2021	Community
EPI_ISL_5059468	9/29/2021	University
EPI_ISL_5059451	9/29/2021	University
EPI_ISL_5059685	9/29/2021	University
EPI_ISL_5059682	9/29/2021	Community
EPI_ISL_5059609	9/30/2021	University
EPI_ISL_5059624	9/30/2021	University
EPI_ISL_7455920	9/30/2021	Community
EPI_ISL_5059671	9/30/2021	Community
EPI_ISL_5091546	9/30/2021	Community

EPI_ISL_5059583	9/30/2021	University
EPI_ISL_5059268	9/30/2021	Community
EPI_ISL_5059654	9/30/2021	University
EPI_ISL_5059059	10/1/2021	University
EPI_ISL_5059394	10/1/2021	University
EPI_ISL_5059600	10/1/2021	University
EPI_ISL_5059449	10/1/2021	University
EPI_ISL_5059107	10/1/2021	Community
EPI_ISL_5091882	10/1/2021	Community
EPI_ISL_5091881	10/1/2021	Community
EPI_ISL_5059536	10/1/2021	Community
EPI_ISL_5059676	10/1/2021	University
EPI_ISL_8365613	10/2/2021	Community
EPI_ISL_5418284	10/3/2021	Community
EPI_ISL_5193299	10/4/2021	University
EPI_ISL_5328573	10/4/2021	University
EPI_ISL_5193327	10/4/2021	Community
EPI_ISL_5193291	10/4/2021	University
EPI_ISL_5193359	10/4/2021	University
EPI_ISL_5193349	10/4/2021	University
EPI_ISL_5193294	10/4/2021	University
EPI_ISL_5193287	10/4/2021	University
EPI_ISL_5193289	10/4/2021	Community
EPI_ISL_5193282	10/4/2021	University
EPI_ISL_6650054	10/4/2021	Unknown
EPI_ISL_6058716	10/4/2021	Unknown
EPI_ISL_5193423	10/4/2021	University
EPI_ISL_5193401	10/5/2021	University
EPI_ISL_5328579	10/5/2021	University
EPI_ISL_5193308	10/5/2021	University
EPI_ISL_5328665	10/6/2021	University
EPI_ISL_5328760	10/6/2021	University
EPI_ISL_5328694	10/6/2021	University
EPI_ISL_5328672	10/6/2021	University
EPI_ISL_5328761	10/6/2021	Community
EPI_ISL_5328602	10/6/2021	University
EPI_ISL_5872308	10/6/2021	Unknown
EPI_ISL_5328559	10/6/2021	University
EPI_ISL_5328606	10/7/2021	University
EPI_ISL_5328663	10/7/2021	University
EPI_ISL_5328584	10/7/2021	University
EPI_ISL_5328784	10/8/2021	University
EPI_ISL_5875053	10/10/2021	Unknown

EPI_ISL_5928622	10/10/2021	Unknown
EPI_ISL_5461710	10/11/2021	Community
EPI_ISL_5928904	10/11/2021	Community
EPI_ISL_5308481	10/11/2021	Community
EPI_ISL_5461661	10/11/2021	University
EPI_ISL_6072970	10/11/2021	Unknown
EPI_ISL_5461692	10/12/2021	University
EPI_ISL_5461623	10/12/2021	Community
EPI_ISL_6077336	10/12/2021	Unknown
EPI_ISL_5628793	10/13/2021	Community
EPI_ISL_5628667	10/13/2021	University
EPI_ISL_5628771	10/13/2021	University
EPI_ISL_5628798	10/13/2021	Community
EPI_ISL_5628858	10/13/2021	Community
EPI_ISL_5628821	10/13/2021	University
EPI_ISL_5628840	10/13/2021	University
EPI_ISL_5628730	10/14/2021	Community
EPI_ISL_5628778	10/14/2021	Community
EPI_ISL_5628761	10/14/2021	University
EPI_ISL_5628696	10/14/2021	University
EPI_ISL_5628679	10/14/2021	University
EPI_ISL_5628731	10/14/2021	Community
EPI_ISL_5628809	10/14/2021	Community
EPI_ISL_6077970	10/14/2021	Unknown
EPI_ISL_8365799	10/15/2021	Community
EPI_ISL_5644184	10/15/2021	Community
EPI_ISL_5644508	10/15/2021	Community
EPI_ISL_5644384	10/15/2021	Community
EPI_ISL_7658986	10/15/2021	Community
EPI_ISL_7659086	10/15/2021	Unknown
EPI_ISL_7659247	10/15/2021	Unknown
EPI_ISL_7659339	10/15/2021	Unknown
EPI_ISL_5644381	10/18/2021	University
EPI_ISL_5644468	10/18/2021	Community
EPI_ISL_5644515	10/18/2021	Community
EPI_ISL_5644512	10/18/2021	Community
EPI_ISL_5644507	10/18/2021	Community
EPI_ISL_5644506	10/18/2021	Community
EPI_ISL_5644178	10/18/2021	Community
EPI_ISL_5644488	10/18/2021	University
EPI_ISL_5644485	10/18/2021	Community
EPI_ISL_5644119	10/18/2021	Community
EPI_ISL_5644352	10/18/2021	Community

EPI_ISL_5644203	10/18/2021	University
EPI_ISL_5644236	10/18/2021	University
EPI_ISL_5644471	10/18/2021	University
EPI_ISL_5644346	10/18/2021	University
EPI_ISL_5779352	10/19/2021	University
EPI_ISL_5644514	10/19/2021	Community
EPI_ISL_5644454	10/19/2021	Community
EPI_ISL_5779254	10/19/2021	Community
EPI_ISL_5779180	10/19/2021	Community
EPI_ISL_5779193	10/19/2021	Community
EPI_ISL_5644495	10/19/2021	Community
EPI_ISL_5644145	10/19/2021	Community
EPI_ISL_5779248	10/20/2021	Community
EPI_ISL_5779408	10/20/2021	Community
EPI_ISL_5779190	10/20/2021	Community
EPI_ISL_5779175	10/20/2021	Community
EPI_ISL_5779234	10/20/2021	Community
EPI_ISL_5779229	10/21/2021	Community
EPI_ISL_5779169	10/21/2021	University
EPI_ISL_5779170	10/21/2021	University
EPI_ISL_6088861	10/22/2021	Unknown
EPI_ISL_5779237	10/23/2021	Community
EPI_ISL_5779311	10/23/2021	Community
EPI_ISL_5779309	10/23/2021	Community
EPI_ISL_5932751	10/23/2021	University
EPI_ISL_5779279	10/23/2021	Community
EPI_ISL_6409546	10/23/2021	Unknown
EPI_ISL_6385920	10/24/2021	Community
EPI_ISL_6419202	10/24/2021	Community
EPI_ISL_7658570	10/25/2021	Community
EPI_ISL_7455973	10/25/2021	Community
EPI_ISL_7745632	10/25/2021	Unknown
EPI_ISL_6389186	10/25/2021	Unknown
EPI_ISL_6075088	10/26/2021	Community
EPI_ISL_7660662	10/26/2021	Community
EPI_ISL_6075064	10/26/2021	Community
EPI_ISL_6403949	10/26/2021	Community
EPI_ISL_6386984	10/26/2021	Community
EPI_ISL_5932871	10/26/2021	Community
EPI_ISL_5932722	10/26/2021	Community
EPI_ISL_5932865	10/26/2021	Community
EPI_ISL_6074999	10/27/2021	Community
EPI_ISL_6390891	10/27/2021	Unknown

EPI_ISL_6074818	10/28/2021	Community
EPI_ISL_6074876	10/28/2021	Community
EPI_ISL_6545612	10/30/2021	Unknown
EPI_ISL_6228210	11/1/2021	Community
EPI_ISL_7379256	11/1/2021	Community
EPI_ISL_6228239	11/1/2021	Community
EPI_ISL_6561174	11/1/2021	Unknown
EPI_ISL_6558701	11/1/2021	Unknown
EPI_ISL_6562721	11/1/2021	Unknown
EPI_ISL_8366075	11/2/2021	Community
EPI_ISL_6228241	11/2/2021	Community
EPI_ISL_6228204	11/2/2021	Community
EPI_ISL_6535423	11/3/2021	Community
EPI_ISL_6584160	11/3/2021	University
EPI_ISL_6535455	11/3/2021	Community
EPI_ISL_6584187	11/4/2021	Community
EPI_ISL_9040177	11/8/2021	Community
EPI_ISL_7660721	11/8/2021	Community
EPI_ISL_6573153	11/8/2021	Community
EPI_ISL_6573042	11/8/2021	Community
EPI_ISL_6666945	11/9/2021	University
EPI_ISL_8366091	11/9/2021	Community
EPI_ISL_7660660	11/9/2021	Community
EPI_ISL_7660657	11/9/2021	Community
EPI_ISL_6666982	11/9/2021	Community
EPI_ISL_6666961	11/9/2021	Community
EPI_ISL_6666830	11/9/2021	Community
EPI_ISL_6666829	11/9/2021	Community
EPI_ISL_6666918	11/9/2021	Community
EPI_ISL_6666825	11/9/2021	University
EPI_ISL_7911193	11/9/2021	Unknown
EPI_ISL_6792129	11/10/2021	Unknown
EPI_ISL_6792105	11/10/2021	Unknown
EPI_ISL_7660714	11/12/2021	Community
EPI_ISL_7410697	11/12/2021	Community
EPI_ISL_7931526	11/12/2021	Community
EPI_ISL_6863159	11/13/2021	Community
EPI_ISL_6901039	11/13/2021	Community
EPI_ISL_6901037	11/13/2021	Community
EPI_ISL_6863133	11/15/2021	Community
EPI_ISL_6863214	11/15/2021	Community
EPI_ISL_6863155	11/15/2021	Community
EPI_ISL_6863247	11/15/2021	Community

EPI_ISL_6863288	11/15/2021	Community
EPI_ISL_6863278	11/15/2021	Community
EPI_ISL_7247988	11/15/2021	Unknown
EPI_ISL_7660735	11/16/2021	Community
EPI_ISL_7120434	11/16/2021	Community
EPI_ISL_7120437	11/16/2021	Community
EPI_ISL_7286078	11/18/2021	University
EPI_ISL_7660790	11/19/2021	Community
EPI_ISL_7499104	11/20/2021	Unknown
EPI_ISL_7269450	11/21/2021	Community
EPI_ISL_7983645	11/23/2021	Community
EPI_ISL_7983791	11/23/2021	Community
EPI_ISL_7945198	11/26/2021	Unknown
EPI_ISL_7735419	11/27/2021	Community
EPI_ISL_7191953	11/27/2021	Community
EPI_ISL_7191950	11/27/2021	Community
EPI_ISL_7192028	11/27/2021	Community
EPI_ISL_8319566	11/29/2021	Unknown
EPI_ISL_7767632	11/29/2021	Unknown
EPI_ISL_8319353	11/29/2021	Unknown
EPI_ISL_7462503	12/1/2021	Community
EPI_ISL_7462655	12/1/2021	Community
EPI_ISL_7495910	12/1/2021	Community
EPI_ISL_7642342	12/2/2021	Community
EPI_ISL_7495926	12/3/2021	Community
EPI_ISL_7495845	12/3/2021	Community
EPI_ISL_7495771	12/3/2021	Community
EPI_ISL_7642844	12/4/2021	Community
EPI_ISL_7642487	12/4/2021	Community
EPI_ISL_7644243	12/6/2021	University
EPI_ISL_7644091	12/6/2021	Community
EPI_ISL_7776757	12/9/2021	University
EPI_ISL_7776764	12/9/2021	Community
EPI_ISL_7776796	12/9/2021	Community
EPI_ISL_8077231	12/11/2021	Unknown
EPI_ISL_7898209	12/13/2021	Community
