# Supplementary material for: One health genomic surveillance and response to a university-based outbreak of the SARS-CoV-2 Delta AY.25 lineage, Arizona, 2021
Source: PLoS One. 2022 Oct 31;17(10):e0272830. doi: 10.1371/journal.pone.0272830 (PMC9621446; doi:10.1371/journal.pone.0272830)
Supplement: Attachment — Submitted filename: Response to Reviewers.docx [file pone.0272830.s002.docx]

August 22, 2022

Dear Editors and Reviewers of PLOS One:

Thank you for the thoughtful review and comments on manuscript PONE-D-22-21082, which describes the “One Health Genomic Surveillance and Response to a University-Based Outbreak of the SARS-CoV-2 Delta AY.25 Lineage, Arizona, 2021”. We greatly appreciate the opportunity to revise our manuscript and submit a revision to PLOS ONE.

This document serves as the rebuttal letter, where we have outlined and responded to each point raised by the academic editor and reviewer(s). Any edits, revisions, and additions that directly impact the manuscript text are indicated as such.

Additionally, we have addressed the additional requirements highlighted by the journal. These are also indicated below for completeness of our manuscript revision.

**Journal Requirements**

**Comment**

Please ensure that your manuscript meets PLOS ONE's style requirements, including those for file naming.

**Response**

The formatting of the manuscript and naming of files has been corrected to meet PLOS ONE’s style requirements. The figures were also uploaded to PACE. The authors would be happy to address any additional formatting changes if something was missed.

**Comment**

Thank you for stating the following financial disclosure: "The NARBHA Institute and the Arizona Department of Health Services (AZDHS) provided funding support for this work. " At this time, please address the following queries:

a) Please clarify the sources of funding (financial or material support) for your study. List the grants or organizations that supported your study, including funding received from your institution.

b) State what role the funders took in the study. If the funders had no role in your study, please state: “The funders had no role in study design, data collection and analysis, decision to publish, or preparation of the manuscript.”

c) If any authors received a salary from any of your funders, please state which authors and which funders.

d) If you did not receive any funding for this study, please state: “The authors received no specific funding for this work.”

**Response**

The NARBHA Institute (#AOTHNARBHA3640C) and the Arizona Department of Health Services (#CTR049427) provided financial support for this work, primarily supporting the genomic sequencing efforts. These agencies had no role in study design, data collection and analysis, decision to publish, or preparation of the manuscript. No authors received a salary from any of the funders. This amended language has been added to the cover letter.

**Comment**
Thank you for stating the following in the Acknowledgments Section of your manuscript: "We also wish to thank the NARBHA Institute and the Arizona Department of Health Services (AZDHS) for funding support for this work." We note that you have provided funding information that is not currently declared in your Funding Statement. However, funding information should not appear in the Acknowledgments section or other areas of your manuscript. We will only publish funding information present in the Funding Statement section of the online submission form. Please remove any funding-related text from the manuscript and let us know how you would like to update your Funding Statement.

**Response**

We have removed this language from the acknowledgement section in the manuscript. The funding statement should read as follows “The NARBHA Institute (#AOTHNARBHA3640C) and the Arizona Department of Health Services (#CTR049427) provided financial support for this work”. This amended language has been added to the cover letter.

**Comment**
Please include your full ethics statement in the ‘Methods’ section of your manuscript file. In your statement, please include the full name of the IRB or ethics committee who approved or waived your study, as well as whether or not you obtained informed written or verbal consent. If consent was waived for your study, please include this information in your statement as well.

**Response**

The full ethics statement has now been included in the methods section of the manuscript. TGen’s Office of Research Compliance & Quality Management (ORCQM) has determined that this project, which was carried out in collaboration with public health partners, is not considered Human Subjects Research.

**Comment**
Please include captions for your Supporting Information files at the end of your manuscript, and update any in-text citations to match accordingly.

**Response**

We have added captions for the tables, figures and supporting information file at the end of the manuscript and updated in-text citations accordingly.

**Comment**

Please review your reference list to ensure that it is complete and correct. If you have cited papers that have been retracted, please include the rationale for doing so in the manuscript text or remove these references and replace them with relevant current references.

**Response**

The references list has been reviewed by the authors and it is complete and correct. We do not have any cited papers to retract.

**Reviewer #1**

**Comment**

Please cut short the abstract mainly the background information. The background information could be limited to 2-3 lines followed by the aim of the study and the results obtained.

**Response**

We have revised the abstract and removed some of the background information.

**Comment**
Is it possible to insert a graphical abstract to grab the attention of the readers/researchers?

**Response**

Thank you for this comment and suggestion. The authors do not have experience in developing a graphical abstract and believe the text, which has now been condensed represents the investigation and outcomes well. We therefore kindly prefer to keep the abstract in text form.

**Comment**
Was there any method blank, extraction blank?

What about positive and negative controls?

What QA/QC measures taken into consideration?
Was the LoD determined?
Was any inhibition noticed?
Were there any replicates for the analysis?

**Response**

We are very grateful to the reviewer for raising these important questions. The authors chose to combine the above questions for the purpose of this rebuttal and address them together in our response below. Furthermore, it was unclear if the reviewer was referring to the COVID-19 testing of human samples (data from which was only addressed briefly in the manuscript) and viral genome sequencing, or the wastewater testing. Therefore, we addressed both.

The original positive clinical samples were identified in multiple CLIA-certified clinical laboratories with FDA allowed tests. The SARS-CoV-2 genomes utilized in the phylogenetic analysis and associated with the outbreak presented here were produced by several different public health, academic, and private laboratories conducting sequencing of COVID-19 positive human samples as described in the methods. Quality control measures, including extraction, positive and negative controls, were employed during the genomic sequencing processes, although they may have varied by each laboratory. Reference 27, Folkerts, et al. (2021) details the sequencing methods utilized in Arizona during the pandemic. There was no inhibition noted.

With respect to replicates for the analysis, sequencing workflows and pipelines do not typically include the generation of duplicate sequences. Only high-quality SARS-CoV-2 genomes with >70% breadth of coverage was included in the analysis and all genomic data presented in this work were obtained from a publicly available dataset hosted by GISAID. The data used in the present work are contained in S1 file or available upon reasonable request to the authors if others wish to repeat the analysis.

The responses to these questions for the wastewater testing are below.

Was there any method blank, extraction blank?

Lines 181-4: We included three reagents blanks each time we process samples, from concentration through RT-qPCR.

What about positive and negative controls?

Lines 181-4: We included three no-template controls and the IDT 2019-nCoV_N Positive Control plasmid (catalog number: 10006625) at dilutions of 1:10, 1:100, and 1:1000 in triplicate, for a total of nine wells containing the positive control.

What are the QA/QC measures taken into consideration?

Lines 181-4: Our standard quality control measures were applied, including embedded reagents blanks(n=3), no-template controls (n=3), and positive controls (IDT 2019-nCoV_N Positive Control plasmid, catalog number: 10006625, at dilutions of 1:10, 1:100, and 1:1000).

Was the LoD determined?

While we have not independently determined the LOD of the CDC N1 assay, the LOD was determined, as part of the FDA EUA process, to be approximately 1 copy per uL with a mean CT of 35.4 (citation below). We have run the N1 RT-qPCR assay on control dilutions with just 20 copies per uL and have consistently had positive results.

Centers for Disease Control and Prevention. CDC 2019-novel coronavirus (2019-nCoV) real-time RT-PCR diagnostic panel [cited 2020 Mar 1]. <https://www.fda.gov/media/134922/download>

Was any inhibition noticed?

We did not test for inhibition.

Were there any replicates for the analysis?

All wastewater samples were tested in triplicate, and those that were selected for sequencing produced a mean CT of less than or equal to 35.

**Comment**
Please incorporate statistical analysis.

**Response**
While the authors agree that statistical analyses are critical in scientific research and appreciate the reviewer’s suggestion to include, the work presented here was conducted as part of a collaborative public health investigation in response to an outbreak of SARS-CoV-2. No statistics, beyond the phylogenetic analyses, were completed.

The main goal of the manuscript is to highlight how genomic and wastewater surveillance data was used to better characterize and provide context to the outbreak that unfolded during the Fall 2021 semester. Furthermore, this work showcases the importance of collaboration and communication between universities, local public health, and other partner entities to guide the overall public health response to an infectious disease outbreak.

Overall, statical analysis may not be truly warranted given that this manuscript is meant to be more descriptive in nature (versus an actual research study) and discuss the various steps taken to respond to the outbreak. Lastly, the authors believe that the epidemiologic and exposure data for the COVID-19 positive cases associated with the university and community is not complete enough to run any robust analyses.

**Comment**
Conclusion section is missing

**Response**

The Public Health Implications section was meant to serve as the conclusions section. We have edited the sub-heading to reflect this.

**Comment**
Please increase the font size in the figures.

**Response**
The font size in the figures has been increased.

We strived to address the comments and suggestions of the editors and reviewers and believe our manuscript is suited for this journal. The authors thank you again for the consideration of our revised manuscript to PLOS ONE.

Sincerely,

Hayley D. Yaglom

**Hayley D. Yaglom, MS, MPH**

One Health Genomics Epidemiologist

Pathogen & Microbiome Division

Translational Genomics Research Institute (TGen North)

3051 W. Shamrell Blvd Ste. 106

Flagstaff, AZ 86005

928-213-6986 | hyaglom@tgen.org
